# Tobacco and tobacco branding in films most popular in the UK from 2009 to 2017

**DOI:** 10.1136/thoraxjnl-2020-214743

**Published:** 2020-09-17

**Authors:** Alexander Barker, Jo Cranwell, Iona Fitzpatrick, Kathy Whittamore, Khaldoon Alfayad, Amira Haridy, Rachael Murray, John Britton

**Affiliations:** 1 Division of Epidemiology and Public Health, University of Nottingham, Nottingham, UK; 2 Department for Health, University of Bath, Bath, UK; 3 Tobacco Control Research Group (partner in Stopping Tobacco Organisations and Products), Department for Health, University of Bath, Bath, UK

**Keywords:** tobacco control

## Abstract

**Background:**

Exposure to tobacco content in films is a cause of smoking uptake in young people. In an earlier study, we reported that tobacco content occurred in 70% of UK box office films popular between 1989 and 2008. We now report an analysis of tobacco content in a sample of the top grossing UK box office films between 2009 and 2017, and of population exposure resulting from audience exposure to the 2017 films.

**Methods:**

Occurrence of tobacco intervals (actual tobacco use, implied use, appearance of smoking paraphernalia or branding) was measured by 5 min interval coding in the 15 most commercially successful films in the UK in each year from 2009 to 2017. A nationally representative survey was used to estimate population exposure to the top 15 films from 2017.

**Results:**

We coded 3248 intervals from the 135 films. Tobacco content appeared in 245 intervals (8%, 95% CI 7% to 9%) across 56 (41%, 95% CI 33% to 49%) films. Tobacco content occurred in films in all BBFC age ratings, and 36 (64%, 95% CI 51% to 77%) of films containing tobacco imagery were classified as suitable for viewing by people aged under 15 years. Although less prevalent than in our earlier study, there was no evidence of a secular decline in tobacco content during this study period. The top 15 films from 2017 delivered approximately 21.6 (95% CI 21.06–22.14) million tobacco impressions to young people aged 10–18 years in the UK.

**Conclusions:**

Tobacco content continues to appear in UK Box Office films and is widely seen by young people, representing a major driver of smoking uptake.

**Key messages**

**What is the key question?**

- Is tobacco content still prevalent in UK box office films?

**What is the bottom line?**

- Tobacco content continues to appear in UK box office films and is widely seen by young people, representing a major driver of smoking uptake.

**Why read on?**

- We present a content analysis of the annual top 15 grossing films at the UK box office between the years 2009 and 2017.

## Introduction

Smoking is the largest avoidable cause of death and disability in rich countries, killing half of all lifelong smokers^1^ and in 2018 causing an estimated 95 600 deaths and more than half a million hospital admissions in the UK.^2^ Since most smokers in the UK begin regular smoking before reaching the age of 18 years,^3^ identifying and preventing causes of smoking uptake in these young people remain a public health priority.

Exposure to tobacco imagery in film is a recognised cause of smoking uptake,^4–6^ and a meta-analysis of prospective cohort studies has estimated that children exposed to high levels of smoking imagery in film are more than 40% more likely to become smokers than those with little or no exposure.^7^ This exposure would be preventable through the age-classification systems that most countries apply to films if smoking and other tobacco imagery were considered harmful by regulators. In the UK, for example, age classification ratings are provided by the British Board of Film Classification (BBFC), whose mission includes protecting the public, and especially children, from content which might cause harm.^8^ The BBFC provide guidance to families to help them make informed decisions about what films are suitable for children. However, the BBFC does not appear to consider smoking to be harmful. In relation to smoking, BBFC guidelines state only that if smoking features to a significant extent in works which appeal to children, this will be indicated in information provided alongside the age classification and that, despite evidence that the effect of smoking is independent of film character type (‘good guy or bad guy’), classification decisions only take into account promotion or glamorisation of smoking.^9^

We have previously reported that 70% of 300 top-grossing UK cinema films in the years 1989–2008 included tobacco content and that 56% of those containing tobacco were rated as suitable for viewing by children aged under 15 years.^10^ To determine whether tobacco imagery continues to be prevalent in contemporary UK box office films, we now report an analysis of tobacco content in the top-grossing box office films distributed in the UK between 2009 and 2017 and estimate the population reach of this tobacco imagery in terms of UK audience impressions (the estimated number of times tobacco content was seen by an audience) to see how much of this content is being seen by young audience.

## Methods

Tobacco content in the 15 annual top grossing box office films in the UK for the years 2009–2017, identified from the British Film Industry Statistical Year Books,^11^ was measured semiquantitatively using the 5 min interval coding method described previously.^12^ Coding for each film began at the start of each film and continued until the end of the credits. In each interval, tobacco content was recorded in each of the following categories.

- Actual tobacco use: actual observed use of tobacco onscreen by any character, coded as cigarette, cigar, pipe or other (such as water pipe or chewing tobacco).
- Implied tobacco use: any implied tobacco use without any actual use onscreen (eg, holding a cigarette without actual smoking or a comment about going for a cigarette), coded as verbal or non-verbal.
- Tobacco paraphernalia: the presence of tobacco or tobacco-related materials, coded by the type of appearance (including cigarette or other tobacco pack, matches, lighter, ashtray, no smoking or smoking area signs).
- Brand appearance: The presence of clear and unambiguous tobacco branding, including cigarette or other tobacco packs, secondary advertising (advertisements appearing within other programmes) and branded merchandising.
- Any tobacco content: Any of the aforementioned.

For coding purposes, multiple instances of the same category in the same 5 min interval were considered to be single event, while instances that ran into consecutive 5 min periods were coded as separate events. Instances in different categories in the same interval were recorded as different events. Approximately 10% of all films were double coded and any discrepancies were discussed between coders and amended accordingly. Information on the age rating of each film was gained from the BBFC; information on the production of each film was gained from the Internet Movie Database.

To estimate exposure to a sample of films included in our content analysis, we included questions on viewing the 15 annual top grossing box office films in the UK for the year 2017 in a national survey of young people carried out by YouGov PLC. In accordance with YouGov practice, people aged 10–18 years were recruited by direct email invitations to a random sample of panellists from a database of individuals who had consented to be contacted. Consenting respondents then followed a link to an online survey where they were asked to indicate which of the 15 films they had seen. We then combined our estimates of tobacco imagery content in the films seen with UK mid-year population estimates for 2018^13^ to estimate gross and per capita impressions, using previously reported methods.^14^ Dividing gross impressions by population mid-year estimates provided per capita impressions, the estimated number of tobacco impressions delivered to each person.

Coding data were entered directly on a Microsoft Excel (Version 16) spreadsheet as the films were watched and analysed using basic descriptive procedures and regression analysis in the Statistical Package for the Social Sciences (IBM SPSS V.24). Spearman’s correlation was used to assess the relationship between the amount of tobacco intervals per hour of film over time. P values of <0.05 were deemed statistically significant.

## Results

The 135 films analysed totalled 265.2 hours (15 912 min) of film time, with a mean of 117.87 (SD 22.10) min/film, and a range from 82 min (*Secret Life of Pets*) to 174 min (*The Wolf of Wall Street*). The BBFC U, PG, 12/12A, 15 and 18 categories contained 17%, 16%, 50%, 14% and 2% of films, respectively. The majority of films analysed (59%, 95% CI 51% to 67%, 79/135) were produced solely in the USA. UK producers were involved in 25% (34/135) of films and were solely responsible for 3% (95% CI 1% to 5%, 4/135) of films. Other countries were involved in creating 17% (95% CI 11% to 23%, 23/135) of films. Only one film (*Taken 2*) had no UK or US production involvement.

There were 3248 5 min intervals in the films, with a mean of 24 per film, range 17–35. Tobacco content occurred in 245 intervals (8% of the total, 95% CI 7% to 9%) across 56 (41%, 95% CI 33% to 49%) films. The respective proportions of films containing any tobacco intervals in each of the BBFC age categories are shown in table 1.

**Table 1:** Proportion of films in BBFC age category containing tobacco intervals

BBFC age rating*	Proportion of films in each age category containing tobacco intervals
U	1/23 (4%, 95% CI 0% to 12%)
PG	5/22 (23%, 95% CI 5% to 41%)
12/12A	30/68 (44% 95% CI 32% to 56%)
15	17/19 (89% 95% CI 75% to 100%)
18	3/3 (100%, 95% CI 100% to 100%)

*U, suitable for all ages; PG, parental guidance; 12/12A, suitable for 12 years and over; 15, suitable for 15 years and over; 18, suitable only for adults.

BBFC, British Board of Film Classification.

Tobacco intervals occurred in 40% (95% CI 32% to 48%, 53/132) of all films rated suitable for watching by people aged under 18 years by the BBFC, as did 87% (95% CI 83% to 91% 214/245) of all tobacco intervals. Nearly two-thirds (64%, 95% CI 51% to 77%) of films containing tobacco imagery were classified as suitable for viewing by people aged under 15 years. Tobacco imagery occurred in 39% (95% CI 31% to 47%, 50/129) of films produced with some US involvement, and in all six (100%, 95% CI 100% to 100%) of those produced with no US involvement (p=0.004, Fisher’s exact test).

**Figure 1:**
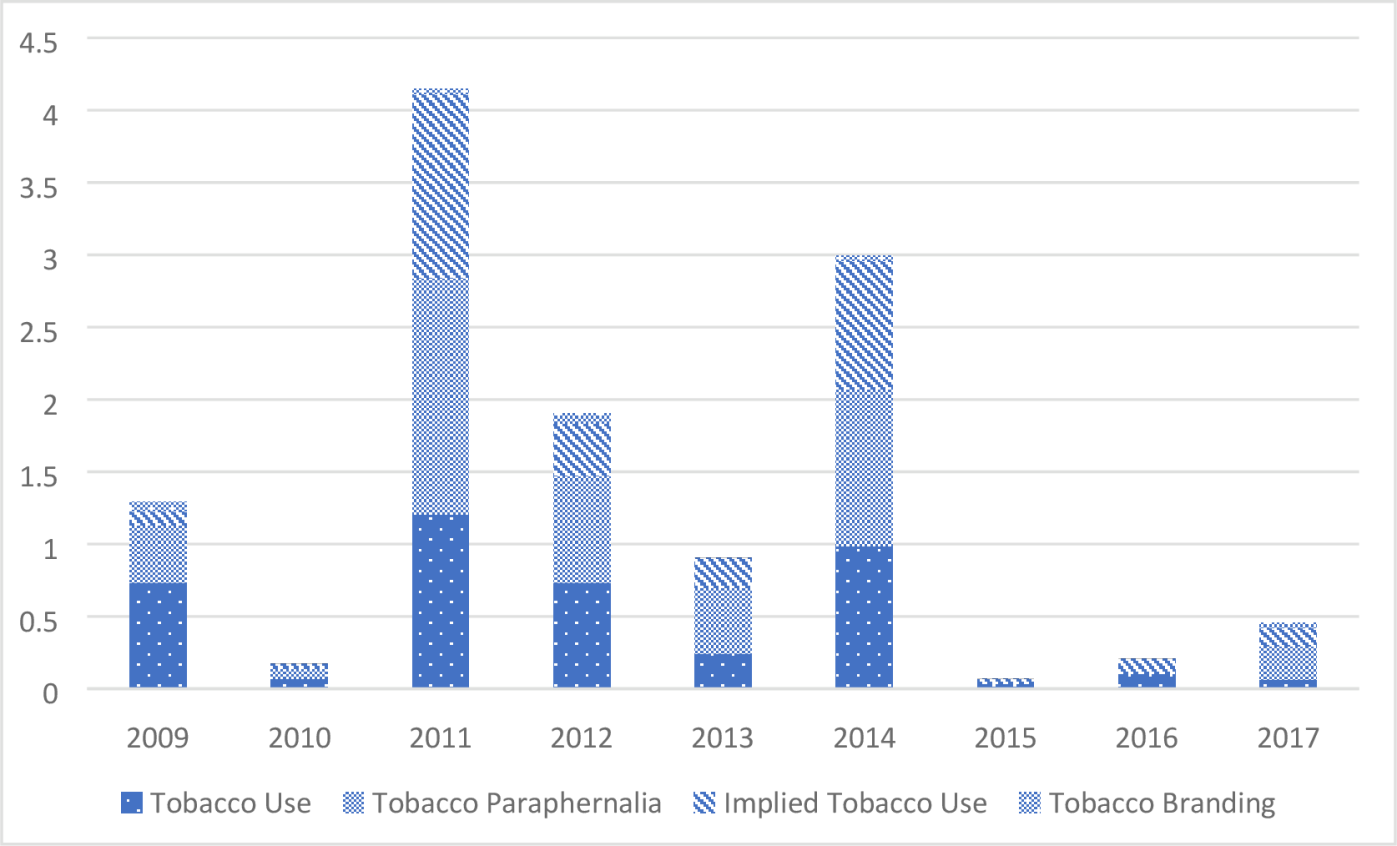
Trends in mean tobacco intervals per hour of film, 2009–2017.

The average number of intervals containing the different categories of tobacco content per hour of film varied from year to year, though not to a statistically significant extent and with no obvious trend. However, the mean number of tobacco intervals per hour of film has been particularly low since 2015 (figure 1).

**Figure 2:**
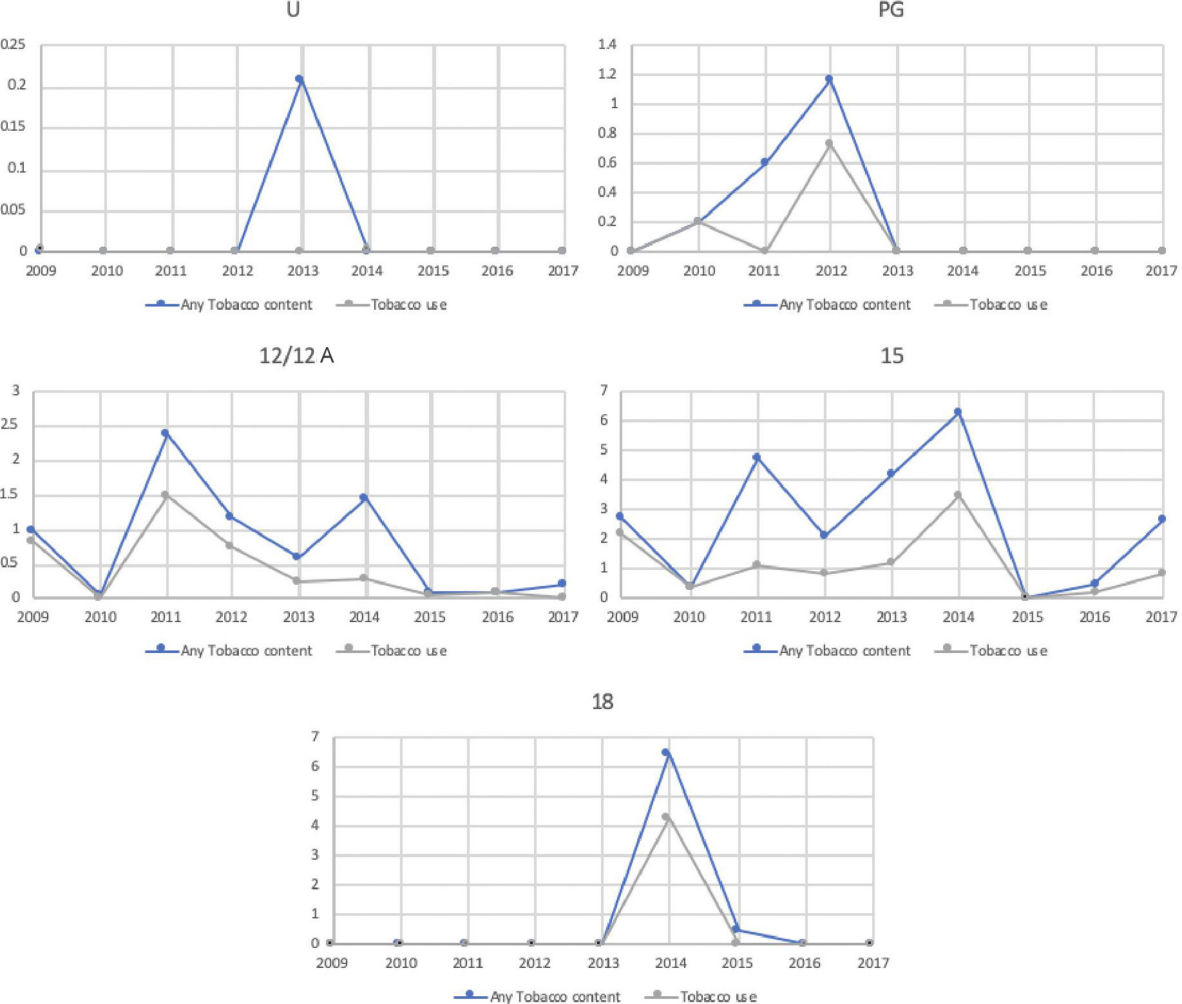
Trends in mean numbers of intervals containing any tobacco imagery, or tobacco use, per hour each year in relation to British Board of Film Classification category. There were no films rated 18 in the top UK box office films for 2009–2013. 12/12A, suitable for 12 years and over; 15, suitable for 15 years and over; 18, suitable only for adults; PG, parental guidance; U, suitable for all ages.

Actual tobacco use occurred in 50% of all tobacco intervals (95% CI 44% to 56%, 123/245), in 24% of films (95% CI 17% to 31%, 32/135) and in a majority of cases (72 intervals, 59%, 95% CI 53% to 65%) involved cigarette smoking. Almost all (31/32) films featuring actual tobacco use were in BBFC 15 and lower categories, and more than half (56%, 95% CI 38% to 73%, 18/32) were rated suitable for audiences age 12 or lower. There was no clear trend in the frequency of tobacco intervals, or of intervals including tobacco use, per hour of film within BBFC age-classification categories, though there was no tobacco use in any U-rated film (figure 2). Tobacco use was much more common in films produced solely in the UK (occurring in 23 of 85 intervals, 28%, 95% CI 18% to 38%) than those produced in the US (49/1850 intervals, 3%, 95% CI 2% to 4%, p<0.001).

Implied tobacco use occurred in 92 intervals (3%, 95% CI 2% to 4%, 92/3248) in 29 films (21%, 95% CI 14% to 28%, 29/135), typically in the form of non-verbal cues (83%, 95% CI 75% to 91%, 72/92). Tobacco paraphernalia occurred in 46 films (34%, 95% CI 26% to 42%, of all films) and in 4% of all intervals (95% CI 3% to 5%, 135/3248), typically in the form of ashtrays (alone or with other paraphernalia; 44% of paraphernalia intervals (95% CI 35% to 52%, 59/135), cigarette or other tobacco packs (12%,95% CI 7% to 17%, 16/135), lighters (18%, 95% CI 17%–31%, 24/135) or matches (7%, 95% CI 3% to 11%, 10/135).

Tobacco branding, typically on tobacco packs, was present in seven intervals in six films. Five of these films were US productions, and one solely UK. *Marlboro* was the only brand to appear in more than one film, with *Marlboro Gold* appearing in two intervals in *Slumdog Millionaire* and *Marlboro* in a single interval in *The Amazing Spiderman*. More than one brand occurred in a single interval in *Men in Black 3* (*Lucky Strike, Embassy*). A fictional brand (*Old* Toby) appeared in one interval in *The Hobbit* and multiple fictional brands (*Wellesley*, *Emperor* and *Carolina Menthol*) in one interval in *IT* (table 2).

**Table 2:** Films containing tobacco branding

Title	Release year	Country of origin	BBFC rating	Branding intervals, n	Brand(s)
*Slumdog Millionaire*	2009	UK	15	2	Marlboro (Gold)
*Hangover I*I	2011	USA	15	1	K&J Lights
*Amazing Spider Man*	2012	USA	12	1	Marlboro
*Men in Black 3*	2012	USA	PG	1	Lucky Strike, Embasssy
*The Hobbit: An Unexpected Journey*	2012	USA/NZ	12	1	Old Toby
*IT*	2017	USA/Canada	15	1	Wellesley, Emperor, Carolina Menthol

BBFC, British Board of Film Classification.

### Trends over time

**Figure 3:**
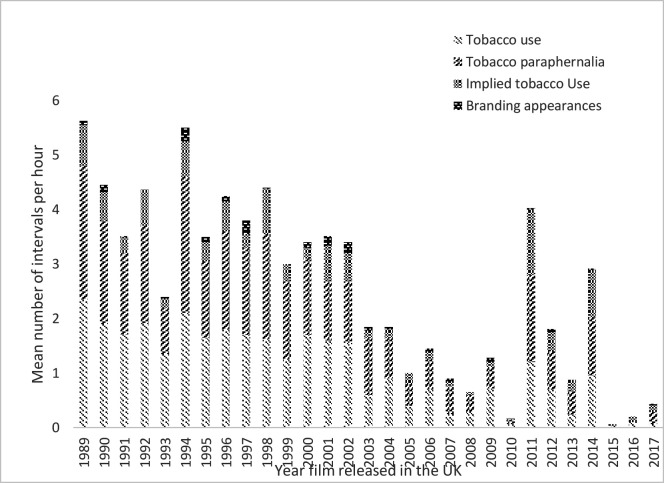
Mean number of intervals containing tobacco content per hour of film, 1989–2017.

When the data from the current study were compared with the data from the previous study, the mean number of tobacco intervals per year are negatively correlated (r(27)=−0.789, p<0.01) (figure 3).

#### UK population exposure

Our YouGov Omnibus survey obtained data on which of the 15 2017 films sampled, four of which included tobacco content, had been seen by a nationally representative sample of 935 young people aged 10–18 years. The film with the most content, *IT*, was rated 15 and was seen by 26% (95% CI 23% to 28%) of young people aged 10–18 years in the UK. Using UK population estimates,^15^ we estimate that the four films delivered 21.6 (95% CI 21.06 to 22.14) million tobacco impressions to young people aged 10–18 years (table 3).

**Table 3:** Estimated gross and per capita tobacco impressions delivered to 10–18 year olds in the UK from the four sampled films from 2017 containing tobacco imagery

Film	Age rating (BBFC)	Gross impressions (million)	95% CI	Per capita impressions	95% CI
*IT*	15	10.53	10.22 to 10.84	1.56	1.51 to 1.61
*Spiderman: Homecoming*	12a	6.21	6.09 to 6.33	0.92	0.90 to 0.94
*Dunkirk*	12a	1.35	1.30 to 1.40	0.2	0.19 to 0.21
*Jumanji: Welcome to the Jungle*	12a	3.51	3.45 to 3.57	0.52	0.51 to 0.53

BBFC, British Board of Film Classification.

## Discussion

This study demonstrates that tobacco content, including tobacco smoking, continues to occur frequently in the most popular UK films; that this content is included in films in almost all age classification groups and that a majority of films containing smoking are classified by the BBFC as suitable for viewing by people aged under 15 years; and that tobacco imagery is significantly more likely to occur in films produced by UK companies. Although the proportion of films containing tobacco, at 40%, is much lower than the 70% we reported in an earlier analysis of films popular between 1989 and 2008,^10^ the proportion of films containing tobacco classified as suitable for viewing by people aged under 15 years was unchanged. Thus, while film makers may have reduced the amount of smoking imagery they include in films over the past decade, BBFC classification policy remains consistently passive in relation to this content,^9^ with classification decisions only taking into account promotion or glamorisation of smoking. Since there is strong causal evidence that exposure to tobacco imagery in films increases smoking uptake in adolescents,^7^ the BBFC thus continues to fail to meet its mission of protecting children from harmful content.^8^

Our study was limited by available coding resources to the top 15 most popular films each year, but these are likely to reflect the prominent pattern of tobacco exposure in films seen in UK cinemas each year since they typically represent around 50% or more of total annual box office takings.^16^ To code content in films, we used a method which has been widely used across a variety of audiovisual media^12 14 16–24^ and used double coding to ensure consistency between coders. Due to the lack of precise viewing figures, a nationally representative YouGov Omnibus survey was used to estimate the number of tobacco impressions delivered to a sample of the UK population. Our population exposure estimate included films from a single year, 2017; therefore, the UK population exposure to tobacco content in UK box office films throughout the study period is thought to be much higher. The amount of tobacco content in films from 2017 was relatively low; our population estimate reflects this and would likely be higher for years with more tobacco content. Our tobacco exposure estimate is for the UK population, but these films are viewed worldwide, and therefore, UK population exposure figures probably represent a very small proportion of the true total global exposure. We used interval coding methods to generate semiquantitative measures of content over a standardised period of time to allow direct comparison between programmes which are shown for different amounts of time, therefore allowing an exploration of the percentage proportion of a programme. This method can lead to both underestimation (if high-frequency appearances are concentrated in short periods of time) and overestimation (if short appearances transition into two intervals) and has been widely used in previous studies.^12 14 17 19–23 25–30^ Alternative approaches such as frequency analysis,^31–35^ whereby all visual appearances are counted as individual events irrespective of duration, are available but assume that a single long appearance carries the same impact as a short appearance. Our estimate of population exposure is also based on 5 min intervals, rather than incidents, and therefore may be lower than estimates based on incidents alone.

While it is promising that tobacco content in films occurred less frequently during the present study period from 2009 to 2017, this does not appear to reflect a secular trend; rather, our findings mirror those reported in US films in which the frequency of tobacco content declined to 2010 and then increased.^36^ Furthermore, even at this lower level of occurrence, this content in UK films generates substantial population exposure, with films in a single year delivering 21.6 million tobacco impressions to young viewers.

It is important to consider that many of these films were also released to an American audience. While there are differences in the age ratings between the UK and the USA, and the way that these ratings prevent young people from viewing content unsuitable for them, 17 films which were rated 15 in the UK were rated higher (‘R’) in the USA. In our population exposure, the film containing the most tobacco content and which delivered the most viewer impressions, *IT* was rated a 15 in the UK and an ‘R’ in the US. If the film had been given an adult (18) rating in the UK, this may have prevented this film from delivering a large proportion of tobacco impressions to young people.

Viewing habits are changing and online video-on-demand (VOD) services such as Netflix and Amazon Prime Instant Video, which allow users to watch whatever they choose at any time of day, are becoming increasingly popular.^37–42^ A number of films included in the present study are now featured on VOD services, thereby increasing exposure to tobacco content found in these films. These changes in the way that film content is consumed make it even more important that film classification authorities such as the BBFC follow WHO guidance, by prohibiting the appearance of branding in films and applying adult classifications to films containing tobacco imagery,^43^ since film makers tailor content carefully to the requirements of their target age rating for each film. Knowing that including tobacco would ensure an adult rating in the global fourth largest national film market^44^ would therefore be likely to result in widespread exclusion of tobacco imagery from all films aiming for a less than adult rating. We concede that due to changing viewing habits, a limitation of the current study is the focus on the top-15 UK box office films released annually in a cinema’s, as these films as viewers can watch films from previous years on VOD services. Furthermore, a number of films and series are released exclusively on VOD services. Future studies should explore films on these services.

The current study did not measure e-cigarette content; as e-cigarettes have become more popular over time, it is likely that this will be reflected in UK box office films. Future studies should explore the changing representation of tobacco products in films.

The current study thus provides further evidence in support of more effective UK implementation of the tobacco promotion policies outlined in the Framework Convention for Tobacco Control^45^ to reduce youth exposure to smoking in movies. It also provides clear evidence that the BBFC has yet to deliver on its mission to protect children from this form of harmful imagery when they visit the cinema. Future tobacco content, whether glamorised or not, should be considered when assigning age classifications to films, and all films containing tobacco content should be assigned an adult (18) rating to protect children from this content.
